# Cost-effectiveness of TLC-NOSF dressings versus neutral dressings for the treatment of diabetic foot ulcers in France

**DOI:** 10.1371/journal.pone.0245652

**Published:** 2021-01-22

**Authors:** Franck Maunoury, Anaïs Oury, Sophie Fortin, Laetitia Thomassin, Serge Bohbot, on behalf of the Explorer Study

**Affiliations:** 1 Health Economics, Statesia, Le Mans, France; 2 Global Regulatory Affairs & Market Access, URGO Medical, Chenôve, France; 3 Global Medical Affairs, URGO Medical, Paris, France; URCEco Ile de France Hopital de l'Hotel Dieu, FRANCE

## Abstract

This study assesses the cost-effectiveness of Technology Lipido-Colloid with Nano Oligo Saccharide Factor (TLC-NOSF) wound dressings versus neutral dressings in the management of diabetic foot ulcers (DFUs) from a French collective perspective. We used a Markov microsimulation cohort model to simulate the DFU monthly progression over the lifetime horizon. Our study employed a mixed method design with model inputs including data from interventional and observational studies, French databases and expert opinion. The demographic characteristics of the simulated population and clinical efficacy were based on the EXPLORER double-blind randomized controlled trial. Health-related quality of life, costs, and resource use inputs were taken from the literature relevant to the French context. The main outcomes included life-years without DFU (LYs_w/DFU_), quality-adjusted life-years (QALYs), amputations, and lifetime costs. To assess the robustness of the results, sensitivity and subgroup analyses based on the wound duration at treatment initiation were performed. Treatment with the TLC-NOSF dressing led to total cost savings per patient of EUR 35,489, associated with gains of 0.50 LY_w/DFU_ and 0.16 QALY. TLC-NOSF dressings were established as the dominant strategy in the base case and all sensitivity analyses. Furthermore, the model revealed that, for every 100 patients treated with TLC-NOSF dressings, two amputations could be avoided. According to the subgroup analysis results, the sooner the TLC-NOSF treatment was initiated, the better were the outcomes, with the highest benefits for ulcers with a duration of two months or less (+0.65 LY_w/DFU_, +0.23 QALY, and cost savings of EUR 55,710). The results from the French perspective are consistent with the ones from the German and British perspectives. TLC-NOSF dressings are cost-saving compared to neutral dressings, leading to an increase in patients’ health benefits and a decrease in the associated treatment costs. These results can thus be used to guide healthcare decisionmakers. The potential savings could represent EUR 3,345 per treated patient per year and even reach EUR 4,771 when TLC-NOSF dressings are used as first line treatment. The EXPLORER trial is registered with ClinicalTrials.gov, number NCT01717183.

## Introduction

Diabetes is a serious progressive disease estimated to affect 422 million adults worldwide. Its prevalence has been steadily increasing over the past decades [1], affecting 5% of the population in France in 2015, representing more than 3.3 million people [2]. Over time, diabetes damages various organs and can result in reduced distal blood flow, which, combined with nerve damage in the feet, increases the occurrence of foot ulcers and infection, eventually requiring limb amputation. It is estimated that 19% to 34% of patients with diabetes mellitus will develop a diabetic foot ulcer (DFU) during their lifetime [3]. These chronic wounds require several months to heal, and their recurrence rate is around 40% within one year after the initial wound closure [3]. According to the largest observational study on DFU management in Europe (The Eurodiale study), more than half of DFUs become infected [4], and moderate or severe DFU infections lead to amputation in approximately 20% of cases [5]. Eventually, the risk of death at five years for a patient with a DFU is reported to be 2.5 times as high as that for a patient with diabetes without an ulcer [6]. DFUs and the associated infections thus constitute a major risk factor for emergency department visits and hospital admission.

In France, while the incidence of foot ulcers in individuals with diabetes, neuropathy, and peripheral arterial disease is estimated to be around 80,000, more than 26,000 hospitalizations for DFU care and 8,400 hospitalizations for lower limb amputation were registered in patients with diabetes in 2016 [7,8]. Besides their consequences on the health and well-being of patients, DFUs also impose a large economic burden on health systems and national economies. In France, EUR 660 million is attributable annually to DFU care, which includes only inpatient care and amputations (without outpatient care) [7].

To enhance the healing of DFUs, decrease the occurrence of their complications, and alleviate the associated economic burden, new drugs and medical devices are regularly developed based on the growing amount of knowledge on the disease and its progression. In particular, a deleterious proteinase imbalance on the wound bed and poor tissue perfusion have been correlated with wound healing delay for DFUs [9–11]. The assessed dressings are based on the Technology Lipido-Colloid with Nano Oligo Saccharide Factor (TLC-NOSF), meaning they are impregnated with a lipido-colloid healing matrix containing sucrose octasulfate potassium salt. This TLC-NOSF healing matrix has been shown to inhibit the excess matrix metalloproteinases present in chronic wounds and to promote the proliferation and migration of endothelial cells [12]. More significantly, the superior healing enhancer properties of TLC-NOSF dressings have been demonstrated through randomized controlled trials (RCTs), first in the management of leg ulcers [13,14] and then in DFU management [15]. In a large, multicenter, double-blind RCT involving five European countries (known as the EXPLORER study), Edmonds et al. [15] demonstrated that treating foot ulcers in persons with diabetes, neuropathy, and peripheral arterial disease using TLC-NOSF dressings significantly increases the wound closure rate and decreases the time to reach wound closure, compared with a neutral dressing. The high-quality evidence in the EXPLORER study established for the first time that a dressing impregnated with a specific matrix can enhance DFU wound healing. Real-life evidence from a pooled analysis of clinical data from eight observational studies on TLC-NOSF dressings in France and Germany [16] was consistent with the healing rates of DFUs and leg ulcers seen in that RCTs. This analysis included more than 10,000 patients with chronic wounds, of whom 7,903 had leg ulcers, 1,011 had pressure ulcers, and 1,306 had DFUs. Moreover, based on the subgroup analysis of French cohorts, time-to-closure also appeared to be significantly shorter for wounds treated with a TLC-NOSF dressing as a first-line treatment compared with those where it had been prescribed only after using another primary dressing. This applied to all wound etiologies, including DFUs (mean time for DFU closure, first-line intervention: 57.5 days [95% IC: 51.4–63.6] versus second-line intervention: 77.3 days [95% CI: 73.6–107.6]). These better clinical outcomes reported when the TLC-NOSF dressings were used in wounds of shorter duration were also consistent with the results of the EXPLORER study [15,17].

To improve DFU prevention and management, the International Working Group on the Diabetic Foot (IWGDF) regularly updates their guidelines, based on systematic reviews of the most recent clinical evidence. Based on the results of their last published review, in 2019, the IWGDF endorsed the superior clinical efficacy of TLC-NOSF dressings and established that there was evidence to support their use in routine care, along with the usual best standard of care (i.e., efficient offloading systems, regular debridement, and appropriate prevention and management of local infection) [18,19]. The National Institute for Health and Care Excellence (NICE) also concluded in their technical guidance that the evidence supports the case for adopting TLC-NOSF dressings to treat DFUs (and venous leg ulcers) under the National Health Service (NHS) because these dressings are associated with increased wound healing compared to non-interactive dressings [20]. The cost-effectiveness of TLC-NOSF dressings has also been demonstrated in the English [20] and German perspectives [21,22].

However, to the best of our knowledge, the evidence on the cost-effectiveness of TLC-NOSF dressings for DFU management in France has not been published yet. This study assessed the cost-effectiveness and cost-utility of TLC-NOSF dressings versus neutral dressings in the treatment of foot ulcers in patients with diabetes from a collective (all payers: compulsory health insurance, supplementary health insurance and patients) French perspective. Additionally, using sub-group analyses, we determine the effects of wound duration at treatment initiation on the results to identify which treatment initiation timing provides the best outcomes. The results of this study could provide some clues for physicians when selecting the most appropriate dressing for patients with DFUs.

## Methods

### Model overview: A Markov microsimulation model (MMM)

To assess the cost-effectiveness and cost-utility of TLC-NOSF dressings in DFU management in France, a discrete-time, state-transition Markov microsimulation model (MMM) has been developed in this study. The base case analysis is based on a previously published and validated model structure [2,23], which has been updated to incorporate more relevant or recent data reflective of the current DFU practices in France. This modeling approach complied with the guidelines of the French National Authority for Health (Haute Autorité de Santé, HAS) [24] and Consolidated Health Economic Evaluation Reporting Standards [25].

Markov models are well recognized for analyzing the clinical and economic consequences of medical decisions, particularly for long-term diseases characterized by repeated risks of events over time [26]. Our MMM consisted of simulating a cohort of 1,000 individuals with DFUs (representative of the average patient) and then following the natural history progression of these DFUs through the various health states over their lifetime horizon. This type of analysis considers the probability of transitioning from one health state to another at regular intervals and makes it possible to count the events that are likely to occur over a selected period of time, depending on the evaluated treatment.

The model structure and data inputs were reviewed by clinical experts in France to ensure local practice patterns were adequately reflected. All data used in the model were derived from published sources.

### The EXPLORER study and compared interventions

The clinical data used in this analysis were supported by the multicenter, double-blind RCT EXPLORER. The protocol and clinical results were previously published in *The Lancet Diabetes & Endocrinology* [15,17,27]. This study was registered on Clinicaltrials.gov (number NCT01717183), received the necessary national and local ethical approvals, and was conducted in compliance with the regulatory requirements of the five countries involved: France, Spain, Italy, Germany, and the UK. The nominative list of the different involved ethical committees was published as supplementary materials of the original publication, and it included notably the Comité de Protection des Personnes (CPP) of Sud Ouest Outre-Mer III (SOOM III) (France), National Reasearch Ethics Committee of Yorkshire (NRES) and the Humber (NREC) (Sheffield) (the UK), Italian Central Ethics Committee, Comitato Etico Azienda Ospedaliero-Universitaria Pisana (Italy), Ethik-Kommission an der Medizinischen Fakultät de Eberhard-Karls-Universität Tübingen und an dem Universitätsklinikum Tübingen (Germany), and Comité de Ética en Investigación Clínica (CEIC) Hospital Clínico San Carlos (Spain) [15].

This multicenter study was conducted in 43 hospitals with specialized diabetic foot clinics, located in the five countries. A total of 240 adult patients (above 18 years old) with an uninfected foot ulcer, diabetes, neuropathy, and peripheral arterial disease were included in the study between March 2013 and March 2016. The primary outcome of the trial was the percentage of wound closures over a 20-week treatment period, analyzed using the intention-to-treat (ITT) analysis. Wound closure was assessed by local investigators blinded to the randomization group, and was defined as 100% epithelialization without exudate, confirmed at least 10 days after closure. Patients were randomly assigned to either the TLC-NOSF treatment group (UrgoStart contact, Laboratoires Urgo, Fr.) or the control group (the same modern contact layer without NOSF, undistinguishable from the TLC-NOSF dressing, and commonly used in DFU management). Both groups were well balanced at the baseline and received a similar standard of care (notably including the same type of offloading, type and frequencies of debridement, local wound care, and secondary dressings) from physicians and caregivers, who were blind to patient allocation. By week 20, 48% of the wounds treated in the TLC-NOSF group achieved wound closure compared to 30% in the control group (odds ratio: 2.60; 95% CI: 1.43–4.73; p = 0.002).

**Fig 1:**
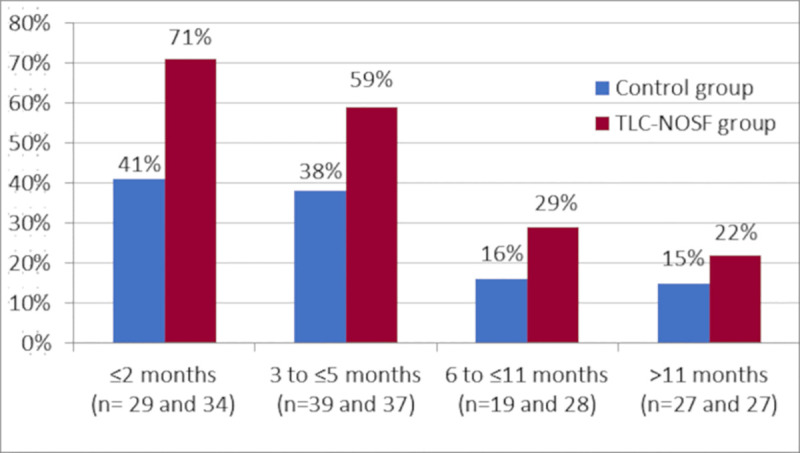
Wound closure rates by week 20 from the EXPLORER study for all patients.

**Fig 2:**
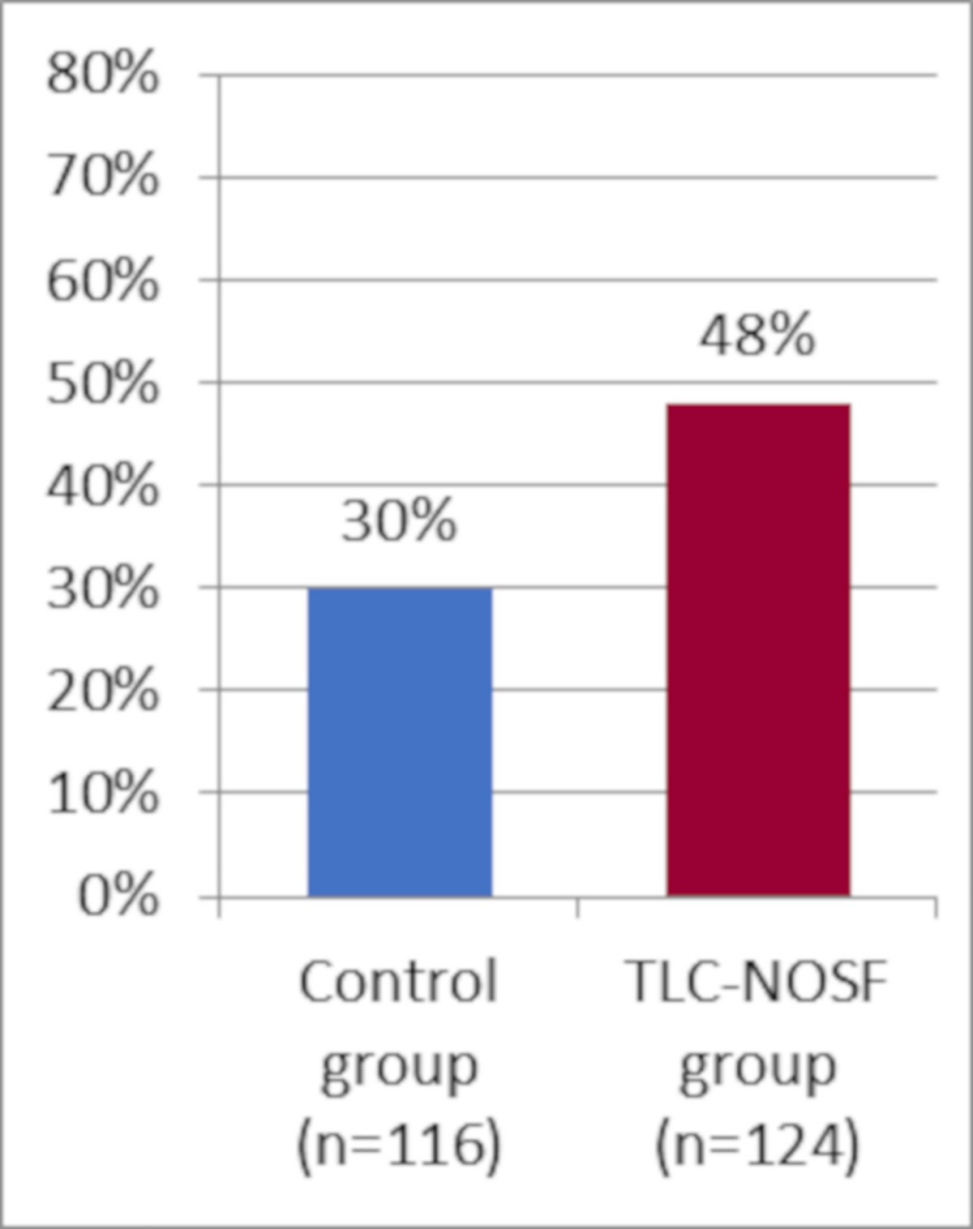
Wound closure rates by week 20 from the EXPLORER study, according to wound duration at treatment initiation.

In France, the 19 centers covered 54 patients (27 treated with TLC-NOSF dressings and 27 with control dressings); however, according to the original regression analysis, the country was not a significant variable in this study. Indeed, in addition to treatment, wound duration was the only other covariate that significantly impacted the wound closure rate (OR: 0.27; 95% CI: 0.15–0.51; p < 0.0001 for closure of wounds with durations of six months and above versus those below six months). The TLC-NOSF consistently achieved better outcomes than the control dressing, regardless of the wound duration reporting the highest difference where the treatment was initiated in wounds with the shortest duration (Figs 1 and 2) [17].

### Baseline characteristics of the simulated population

The baseline characteristics of the simulated population for the base case analysis were generated from the TLC-NOSF group of the EXPLORER study described in Table 1 [15]. Besides the interventions (TLC-NOSF or control dressings), age, sex and DFU duration at baseline were included as impact factors in the cost-effectiveness model: age and sex due to their impact on mortality and DFU duration at baseline due to its impact on the wound closure rate.

**Table 1:** Baseline characteristics of the simulated population for the base case analysis.

Demographics	
Mean age ± SD (years)	64.2 ± 11.2
Age ≥ 70 years (%)	35%
Sex: Men/Women (%)	86%/14%
BMI (kg/m^2^)	30.4 ± 5.7
BMI ≥ 30 kg/m^2^ (%)	48%
**Medical history**	
Diabetes type: Type 2/Type 1 (%)	90%/10%
Mean diagnosed diabetes duration ± SD (years)	17.7 ± 10.3
Mean HbA_1c_ ± SD (%)	7.4 ± 1.3
Hypertension (%)	87%
Mean ABPI value ± SD	0.88 ± 0.24
**DFU characteristics**	
Wound classification (Texas classification)	
IC: superficial ulcer (%)	76%
IIC: ulcer penetrating tendon or capsule (%)	24%
Wound location:	
Plantar	43%
Non-plantar	51%
Wound duration (months)–Median value [Min-max]	5 [1–30]
Wound duration < 6 months (%)	56%
Wound duration ≥ 6 months (%)	44%
Wound surface (cm^2^)–Median value [Min–Max]	[0.5–82.5]

SD: standard deviation, ABPI: ankle-brachial pressure index, BMI: body mass index, IC: ischemic not infected superficial wound, IIC: ischemic not infected wound penetrating.

Data from the EXPLORER study [15].

### Time horizon

The model is based on a time horizon of 40 years and used monthly Markov cycles in accordance with HAS recommendations [24]. This time horizon is long enough to reflect all the expected differences in costs and health effects between the two intervention strategies, as it is the lifetime horizon considering the age of the population at the baseline (64 years). Monthly cycles are relevant in cost-effectiveness and/or cost-utility analyses in the field of DFU management, as they meet the natural history of this medical condition. At the end of each monthly cycle, the model simulates the transition of the patients from one health state to another.

A one- and four-year-time horizons were also used to assess the consistency of the amputation rates generated by our model with the epidemiological data available in France; and additional results, obtained with one- and two-year horizons, are also presented in order to contextualize our results with those obtained from the perspectives of other countries.

### Health states

**Fig 3:**
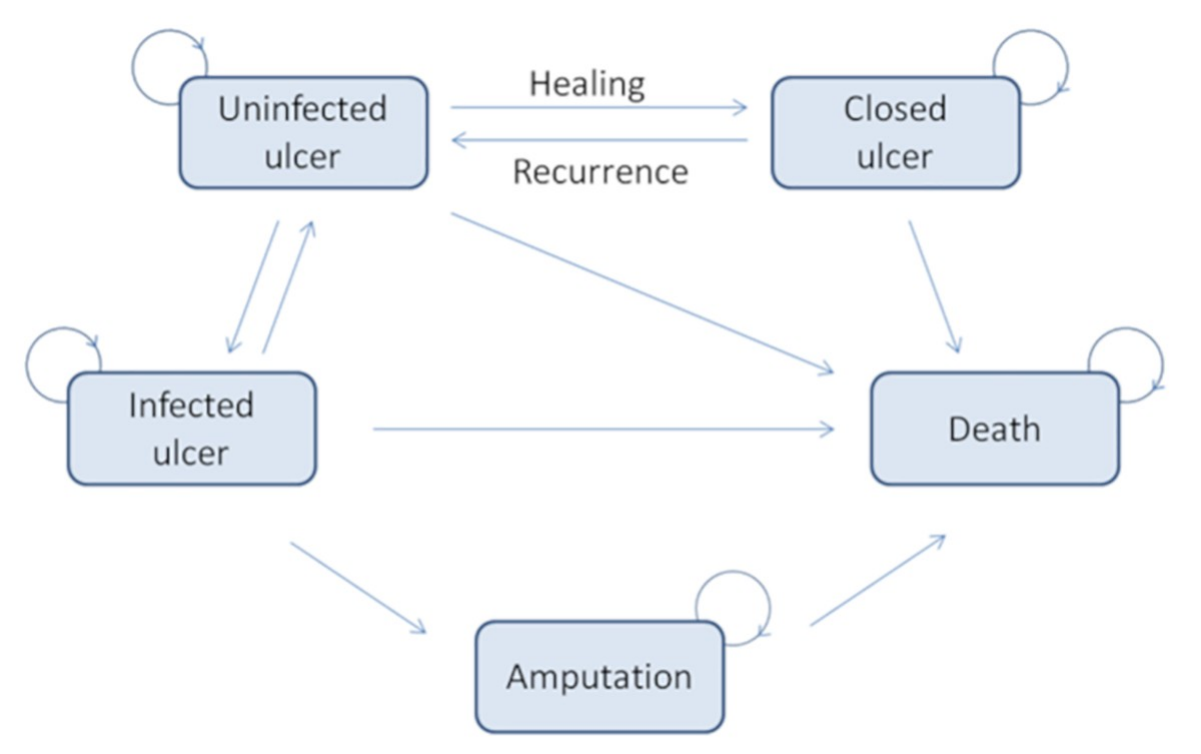
Markov diagram for patients with diabetic foot ulcers.

In our model, five health states have been considered, which include uninfected ulcer, closed ulcer, and the three major complications of the condition: infected ulcer, amputation, and death. These health states are the most commonly used in cost-effectiveness models in DFU [22,28,29] and follow the conservative scenario required by the HAS guidelines [24]. The transitions between these states are illustrated on the Markov diagram (Fig 3).

### Transition probabilities

The monthly transition probabilities between health states were sourced from published studies (Table 2). The probabilities of monthly transition between uninfected and closed ulcer depending on the treatment received were based on the wound healing rate by week 20, as reported in the EXPLORER study (48% in the TLC-NOSF groups and 30% in the control group using ITT analyses) [15]. The probabilities of the other transitions were sourced from Whitehead et al. [28]. The cost-effectiveness model considered all mortality causes according to the published public statistics from the French National Office of Statistics and Economics (INSEE) [30] and the specific mortality related to the different health states of foot ulcers in diabetes, as previously reported in Whitehead et al. [28].

**Table 2:** Monthly transition probabilities.

Monthly transition	Probability
Uninfected ulcer → Closed ulcer	0.091 for the TLC-NOSF group 0.058 for the control group
Closed ulcer → Uninfected ulcer (= recurrence)	0.014
Uninfected ulcer → Infected ulcer	0.036
Infected ulcer → Uninfected ulcer	0.082
Infected ulcer → amputation	0.011
Uninfected ulcer → death	0.009
Infected ulcer → death	0.009
Closed ulcer → death	0.009
Amputation → death	0.120

Data from the EXPLORER study [15] and Whitehead et al. study [28].

### Health and economic outcomes

The health outcomes of the evaluated interventions included life expectancy without ulcer expressed in life years without ulcer (LYs_w/DFU_), life expectancy weighted by health-related quality of life expressed in quality adjusted life years (QALYs), and incidence of amputations over the remaining duration of the patient’s lifetime. The economic outcomes were expressed as expected mean costs over the total lifetime horizon. Aggregated outcomes, aimed to identify the dominant treatment, included the incremental cost-effectiveness ratio (ICER) and incremental cost-utility ratio (ICUR). All outcomes were expressed as discounted results.

### Perspective

The base case analysis adopted a collective perspective (all payers) that considered all stakeholders (compulsory health insurance, supplementary health insurance and patients) concerned with the compared interventions in the French health system. The economic evaluation was made under real-world conditions. The production costs of the interventions studied were identified, measured, and valued independently of their funding source. The health effects were identified and measured from the perspective of the individuals affected by the interventions, based on the outcomes of the EXPLORER study [15]. The utility/preference-based scores used for the valuation of changes in health-related quality of life were obtained from a representative sample of the general population [28,31].

### Cost estimations

Costs were estimated by health state, depending on patient status (inpatient or outpatient) and treatment strategy (TLC-NOSF or control dressings), and are reported in Table 3.

**Table 3:** Monthly costs by health state (input parameters), patient status, and treatment strategy.

Health State	Value for base case analysis TLC-NOSF group	Value for base case analysis Control group
**Outpatient costs**		
Uninfected ulcer	EUR 1,011	EUR 875
Closed ulcer	EUR 0	EUR 0
Infected ulcer	EUR 2,005	EUR 1,840
Amputation	EUR 337	EUR 330
**Inpatient costs**		
Uninfected ulcer	EUR 4,209	EUR 4,188
Closed ulcer	EUR 0	EUR 0
Infected ulcer	EUR 6,516	EUR 6,486
Amputation	EUR 23,560	EUR 23,410
Death	EUR 0	EUR 0

Data from the Whitehead et al. study [28], Cemka 2011 [32], Cnamts 2017 [33], LPPR 2019 [34], NGAP 2019 [35] and the French national reimbursement list for drugs [36].

The resources required for each health state have been identified based on the 2017 report on the resources and products of the French Health Insurance [33], a relevant published study in the French context [28], and a real-life study conducted in France in 2011 including 562 patients with DFUs who were followed by 173 nurses in ambulatory and hospital settings [32]. The reported resources pertain to the same type of patients as in the EXPLORER population (Cnamts 2017: mean age: 69, diabetic patients; Cemka 2011: mean age: 65,8; men: 70%; diabetes type 2: 87%; Whitehead 2011: aged 50–65 years, DFU patients have either type 1 or type 2 diabetes). The data from these references were looked at in depth, and in case of divergence the choice was made on the recommendations of a clinical expert.

Regarding DFU management, the monthly costs for closed ulcers and deceased patients were assumed to equal zero. The monthly costs for uninfected ulcers, infected ulcers, and amputations included offloading devices, dressings, and nurse visits. The costs of biological tests and antibiotics were also added for infected ulcers, costs of surgeries and prostheses for amputations, costs of general and specialist physicians’ consultations for outpatients, and costs of hospital stays for inpatients. The resource valorization was calculated based on the French national reimbursement list for devices *(Liste des Produits et Prestations Remboursables)* [34], the French general classification of health professional activities (*Nomenclature Générale des Actes Professionels*) [35], the French national reimbursement list for drugs [36] for outpatients, and the French national costs scale (*Echelle Nationale des Coûts*) [37] from Whitehead et al. study [28] for inpatients. The detailed resources and their costs are reported in the Supplementary Information S1 File. All costs were expressed in Euro 2019 prices, and, as recommended by the HAS guidelines [24], a 2.5% baseline discount rate was applied to all values, as well as for health effects.

### Utilities

For the cost utility analysis, following the conventional approach, the utility value for death was counted as 0.0. The utility values of the four other health states, as reported in Table 4, were based on published sources relevant in the French context [28,31]. The health states were originally valued by a sample of the general public, using the time trade-off method as recommended by the HAS [24].

**Table 4:** Utility values used in the model.

Health States	Utilities
Uninfected ulcer	0.75
Closed ulcer	0.84
Infected ulcer	0.70
Amputation	0.64
Death	0.00

Data from the Whitehead et al. study [28].

### Model analysis

The model was programmed using Visual Basic Application in Microsoft Excel® 2016 (Microsoft Corp., Redmond, WA) and was fully parameterized to run base case, sensitivity, and subgroup analyses. Visual Basic macros were implemented for the sensitivity analyses.

#### Base case analysis

For the base case analysis, we used a Monte Carlo Markov chain simulation (MCMC) of order 1. The model was run for a specified patient profile, which is described in the Supporting Information S1 File (S1 File). The Markov traces of the cost-effectiveness model for the last simulated individual are shared in the Supporting Information S2 File (S2 File).

#### Sensitivity analyses

To assess the robustness of the results, uncertainty about the model structure and inputs were explored in both deterministic and probabilistic sensitivity analyses, in line with the HAS recommendations [24].

One-way deterministic sensitivity analyses (DSA) were conducted using a change of every input parameter to a minimum and maximum value while keeping all other parameters fixed. The range values of the main parameters of the model patient characteristics at baseline, efficacy of interventions (probability of healing with each dressing), transition probabilities, key unit costs, and each utility value—are reported in Table 5. The demographic and intervention efficacy ranges were based on data from the EXPLORER study [15]. According to the HAS recommendations [24], the unit price of both the TLC-NOSF and control dressings varied from -50% to +20%, and an arbitrary common range was applied to all the other parameters, in this case of [-30%; +30%]. The main results are presented in the form of a Tornado diagram.

**Table 5:** One-way deterministic sensitivity analysis (input parameters).

Values	Base case analysis	Sensitivity analysis [lower bound—upper bound]	
**Demographics of the simulated population**			
Mean age at baseline	64 years old	58 years old	70 years old
Proportion of men	86%	81%	90%
**Probability of healing**			
with TLC-NOSF dressing	0.068	0.048	0.089
*with control dressing*	*0*.*044*	*0*.*031*	*0*.*057*
**Monthly transition probabilities**			
Uninfected ulcer → Closed ulcer			
Healing with TLC-NOSF dressing	0.091	0.064	0.118
Healing with control dressing	0.058	0.041	0.075
Closed ulcer → Uninfected ulcer (= Recurrent ulcer)	0.014	0.010	0.018
*Uninfected ulcer* → *Infected ulcer*	*0*.*036*	*0*.*025*	*0*.*047*
*Infected ulcer* → *Uninfected ulcer*	*0*.*082*	*0*.*057*	*0*.*107*
*Infected ulcer* → *Amputation*	*0*.*011*	*0*.*008*	*0*.*014*
*Uninfected ulcer* → *Death*	*0*.*009*	*0*.*006*	*0*.*012*
*Closed ulcer* → *Death*	*0*.*009*	*0*.*006*	*0*.*012*
*Infected ulcer* → *Death*	*0*.*009*	*0*.*006*	*0*.*012*
*Amputation* → *Death*	*0*.*120*	*0*.*084*	*0*.*156*
**Costs**			
Biological tests	EUR 137	EUR 96	EUR 178
Antibiotics	EUR 458	EUR 321	EUR 596
Mean duration of hospital stays for infected ulcers	6.10 days	4.27 days	7.93 days
Daily cost of hospitalization stays for infected ulcers	EUR 899	EUR 629	EUR 1,169
Amputation surgery	EUR 7,450	EUR 5,215	EUR 9,685
Nurse visit	EUR 14.00	EUR 9.80	EUR 18.20
Unit price for control dressing	EUR 3.04	EUR 1.52	EUR 3.65
Unit price for TLC-NOSF dressing	EUR 7.99	EUR 4.00	EUR 9.59
**Utilities**			
*Uninfected ulcer*	*0*.*750*	*0*.*675*	*0*.*825*
*Closed ulcer*	*0*.*840*	*0*.*756*	*0*.*924*
*Infected ulcer*	*0*.*700*	*0*.*630*	*0*.*770*
*Amputation*	*0*.*640*	*0*.*576*	*0*.*704*

Data from the EXPLORER study [15], Whitehead et al. study [24], Cemka 2011 [32], Cnamts 2017 [33], LPPR 2019 [34], NGAP 2019 [35] and the French national reimbursement list for drugs [36].

To quantify the impact of parameter uncertainty for transition probabilities, the healing rate, and costs on the ICUR of TLC-NOSF dressings compared with control dressings, we performed probabilistic sensitivity analysis (PSA) as a Monte Carlo simulation with 1,000 random samples of the first MCMC sample of 1,000 individuals from the non-informative distribution as a uniform distribution, considering all the available information. The results were reported using the probabilistic cost utility plane and cost utility acceptability curve (CUAC). The CUAC summarizes the uncertainties of the cost utility results, showing the probability for an intervention to be cost effective (defined as an intervention that maximizes the net monetary benefit [NMB] as a function of the willingness-to-pay for a QALY gain).

#### Subgroup analysis

As previously mentioned, according to the original analysis of the EXPLORER clinical trial, wound duration at treatment initiation was, in addition to the evaluated dressings, the only other variable to significantly impact the wound closure rate [15]. Consequently, and in compliance with the HAS expectations, sub-group analysis was conducted depending on the wound duration at baseline (≤ 2 months, 3–5 months, 6–11 months, >11 months). The clinical efficacies of the dressings in each sub-group were extracted from the EXPLORER data [17].

#### Model predictions and validation

The ability of the model to produce consistent results suited to the reality of the decision-making process has been tested. The structure of the model was based on the literature review carried out by Statesia regarding published medico-economic models in DFU patients. As previously mentioned, this structure is equivalent to a simplified version of Whitehead et al.’s model [28], which had been developed for the French context of DFU patient management. Several internal validity tests were then carried out to explore the intrinsic coherence of the model, especially the mathematical or mechanical logic of the relationships between the input parameters and the cost-effectiveness results. The first of these tests consisted in setting equal monthly probabilities of healing for the two tested interventions. No difference in LY_w/DFU_ and QALYs were estimated, which was in line with expectations. To check the relevance of our model predict in terms of amputation, the outcomes have been compared to data from literature. Published data from Santé Publique France (Entred 2007–2010 [38]) indicates that 1.5% of diabetic patients had been amputated in 2010, while our model (with a one-year time horizon) estimates that 1.1% of the patients in the control group were amputated. Moreover, published data from the Institut de veille sanitaire (InVS) in 2015 [39] indicates that 7.5% of the diabetic patients hospitalized in 2010 for a DFU and followed for four years had been amputated, while our model (with a four-year time horizon) indicates that 6% of the patients in the TLC-NOSF group were amputated compared to 8% of the patients in the control group. The results of these comparisons ensure the consistency of the data provided by our model with the French epidemiological data. Regarding external validation, we compared the one and two-year results of our cost-effectiveness model with the results validated by NICE [20] and the German [21] cost-effectiveness models. These results are compared in the Discussion section.

## Results

### Base case analysis

As reported in Table 6, the base case analysis results established that the TLC-NOSF dressing was associated with a gain of 0.5 LYs _w/DFU_, a gain of 0.16 QALYs, and a total cost reduction of EUR 35,489 (uninfected ulcer: -EUR 19,336; infected ulcer: -EUR 15,754; amputation: -EUR 399).

**Table 6:** Life years without DFU, quality-adjusted life years and total costs from the base case analysis (one patient, lifetime horizon, discounted outcomes).

	Life years without DFU (LYs _w/DFU_)	Quality-adjusted life years (QALYs)	Total Costs (EUR)
**TLC-NOSF group**	3.32	4.44	281,360
**Control group**	2.82	4.28	316,849
**Gain of LYs**_w/DFU,_ **QALYs, or total cost reduction**	+0.50	+0.16	-35,489
**ICER (EUR/LYs**_w/DFU_**)** *	Dominant		
**ICUR (EUR/QALYs)** *			

LYs_w/DFU_: life-years without DFU, QALYs: quality-adjusted life-years.

* Dominant means more LYs w/DFU/QALYs at a lower total cost.

The higher number of QALYs achieved for the patients treated with TLC-NOSF dressings was notably related to more frequent health states with better utility value and to a lower mortality due to less frequent DFU complications.

Being less expensive and more effective but also having more utility than the control dressing, the treatment with the TLC-NOSF dressing was established as the dominant strategy for all outcomes compared to the control dressing.

#### Amputation risk

The model also revealed that, for every 100 patients treated with the TLC-NOSF dressing (rather than with the control dressing), two amputations could be avoided (Table 7).

**Table 7:** Amputations avoided for every 100 patients treated with the TLC-NOSF dressing, discounted outcomes.

	TLC-NOSF group	Control group	Amputations avoided with TLC-NOSF dressings
Number of amputations	15/100 patients	17/100 patients	-2/100 patients

### Sensitivity analysis

**Fig 4:**
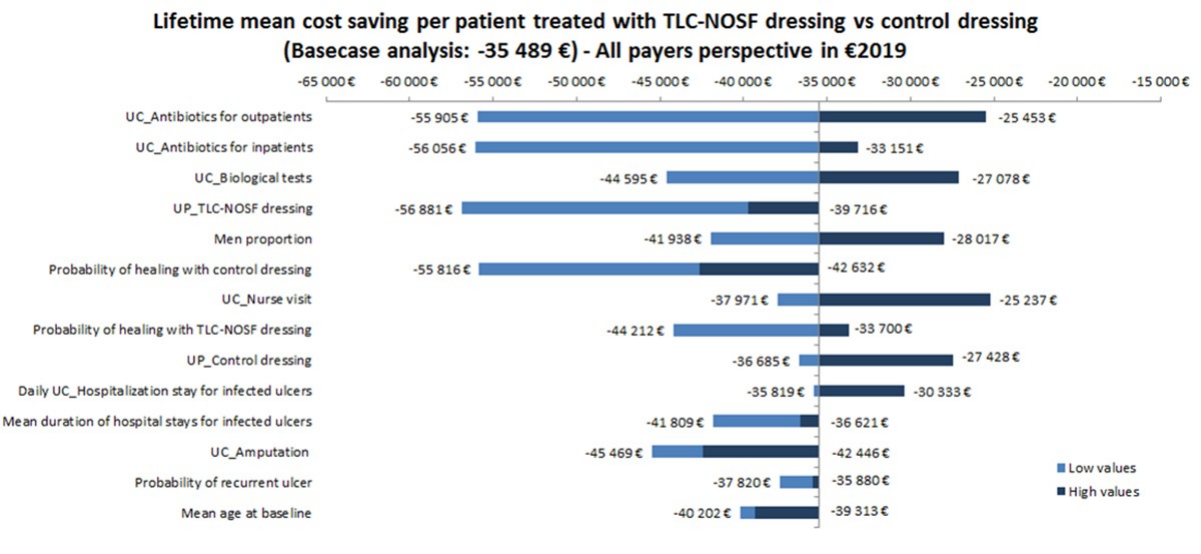
Tornado diagram of key parameters driving the model outcomes. UC_: Unit cost for, UP_: Unit price for, Daily_UC_Hosp: Unit cost per hospitalization day.

According to the one-way deterministic sensitivity analysis, the model outcomes were the most sensitive to the unit cost of antibiotics, unit cost of biological tests, and unit price of TLC-NOSF dressings, with a difference of approximately EUR 17,000 for the latter. The most influential parameters are presented on the Tornado diagram (Fig 4). Since the differences in effectiveness and cost were always in favor of the TLC-NOSF dressings regardless of the tested parameters, as in the base case analysis, it was not necessary to calculate the ICUR to confirm the dominant position of the intervention.

**Fig 5:**
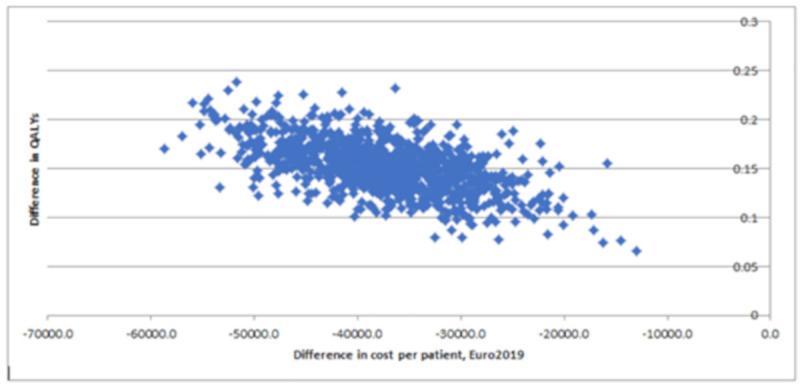
Probabilistic sensitivity analysis: Cost-utility plane for the base case analysis.

**Fig 6:**
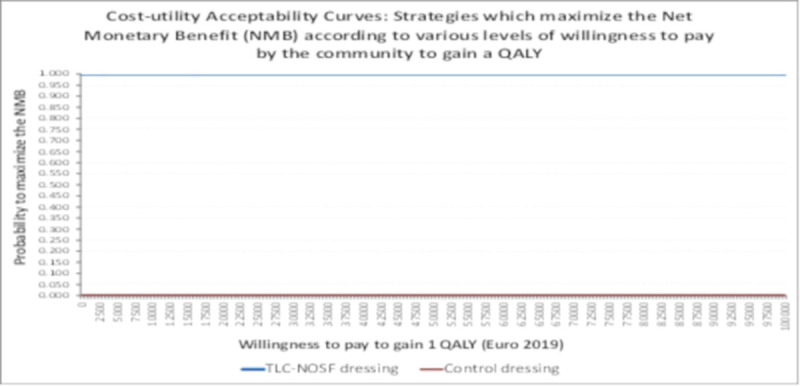
Cost-utility acceptability curves.

The results of the probabilistic sensitivity analysis are illustrated on the cost-utility plane (Fig 5), which describes the difference in QALYs and cost between the TLC-NOSF and control dressing strategies for 1,000 MCMC simulations of 1,000 individuals in each group. All the incremental cost-utility ratios from the simulations consistently confirmed that TLC-NOSF dressings are more effective and less costly than the control dressings. The cost-utility acceptability curves of each dressing strategy were created (Fig 6). The TLC-NOSF dressing strategy maximized the net monetary benefit from a willingness-to-pay of EUR 0.0 per QALY gain. The probability for the TLC-NOSF dressing strategy being cost-effective was consistently higher than that for the control dressing one.

### Sub-group analysis

The subgroup analyses depending on the wound duration at treatment initiation showed the results were consistently in favor of the TLC-NOSF treatment in all subgroups (Table 8). The sooner the TLC-NOSF treatment was initiated, the better the cost-effectiveness and cost-utility outcomes were.

**Table 8:** Life years without DFU, quality-adjusted life years, and total costs depending on wound duration at treatment initiation (one patient, lifetime horizon, discounted outcomes).

	TLC-NOSF group	Control group	Gain of LYs _w/DFU,_ QALYs or total cost reduction
**Life years without DFU (LYs**_**w/DFU**_**)**			
DFU duration: ≤ 2 months	3.12	2.47	+0.65
DFU duration: 3 to ≤ 5 months	2.86	2.41	+0.45
DFU duration: 6 to ≤ 11 months	2.18	1.93	+0.25
DFU duration: > 11 months	1.97	1.92	+0.05
**Quality-adjusted life years (QALYs)**			
DFU duration: ≤ 2 months	4.37	4.14	+0.23
DFU duration: 3 to ≤ 5 months	4.30	4.09	+0.21
DFU duration: 6 to ≤ 11 months	4.03	3.95	+0.08
DFU duration: > 11 months	4.00	3.93	+0.07
**Total costs (EUR)**			
DFU duration: ≤ 2 months	250,579	306,289	55,710
DFU duration: 3 to ≤ 5 months	271,724	311,116	39,392
DFU duration: 6 to ≤ 11 months	327,974	350,010	22,036
DFU duration: > 11 months	344,515	351,223	6,708

**Fig 7:**
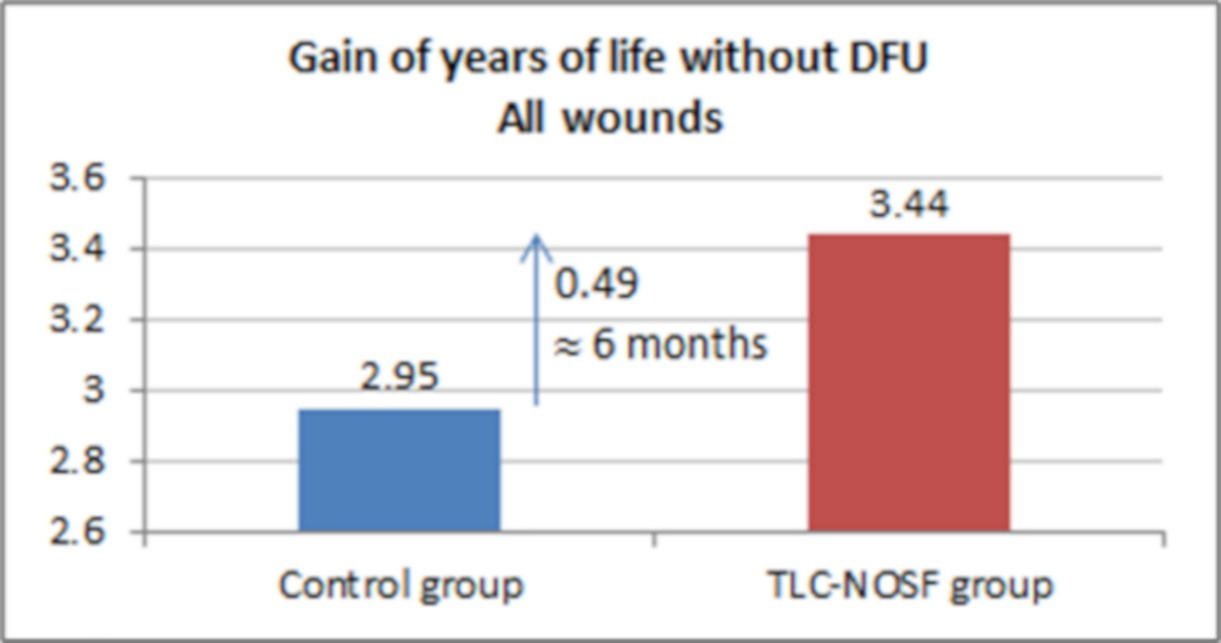
Health benefits: Gain in life years without diabetic foot ulcer for all wounds.

**Fig 8:**
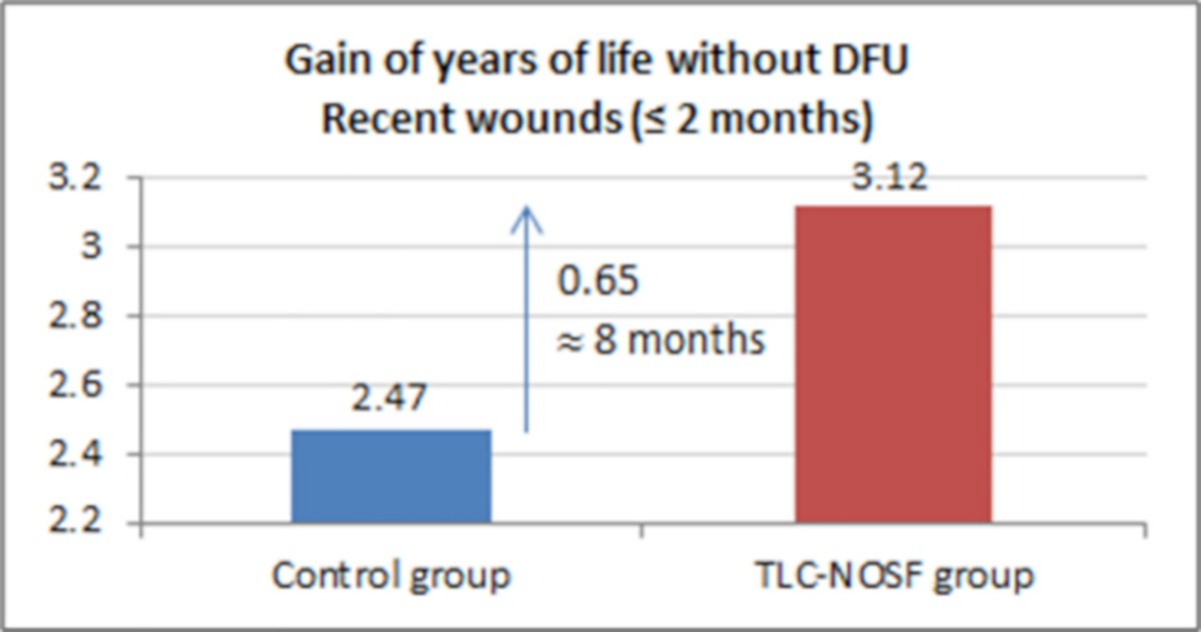
Health benefits: Gain in life years without diabetic foot ulcer for recent wounds (≤ 2 months).

**Fig 9:**
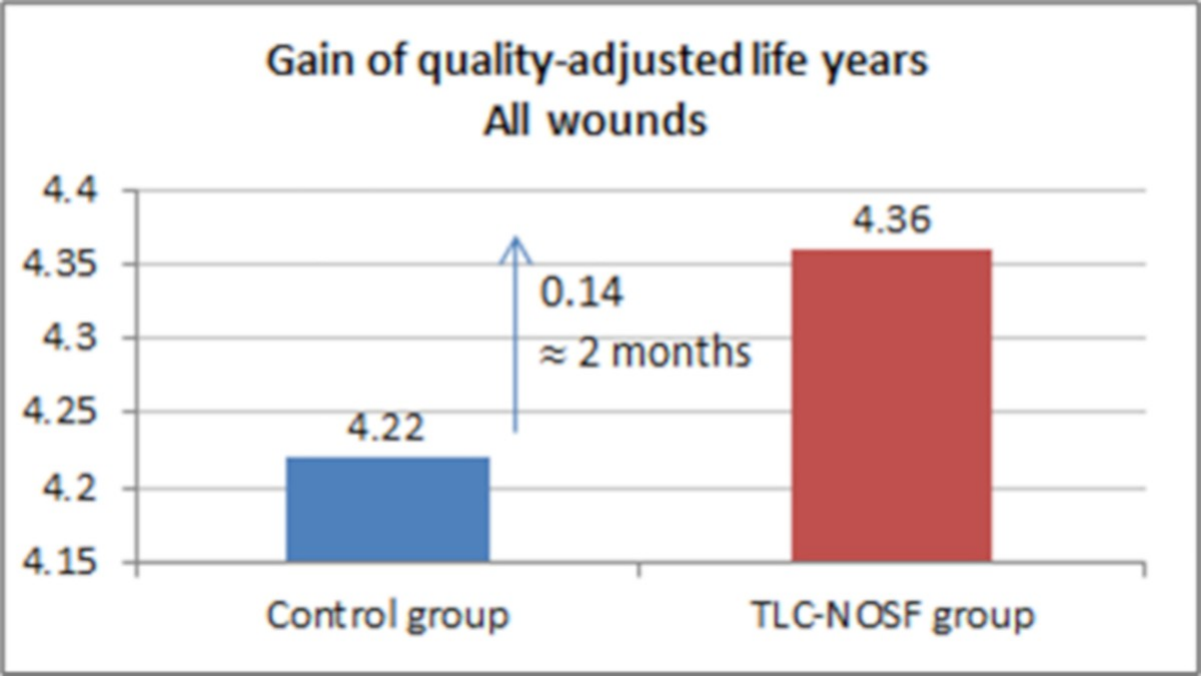
Health benefits: Gain in quality-adjusted life years for all wounds.

**Fig 10:**
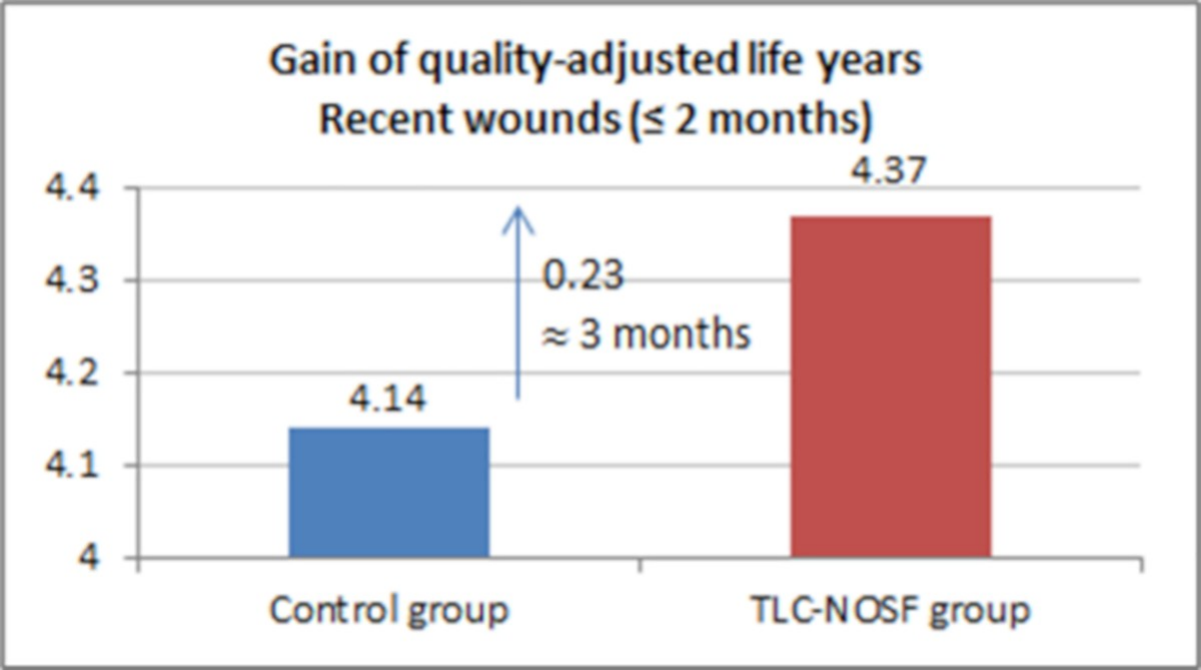
Health benefits: Gain in quality-adjusted life years for recent wounds (≤ 2 months).

The best outcomes were indeed achieved for patients with a DFU lasting two months or less, with gains of approximately eight months without DFU (+0.65 LYs _w/DFU)_ and three months of life adjusted to health-related quality (+0.23 QALYs), in addition to a cost reduction of EUR 55,710 (Figs 7–10).

#### Results with one-year and two-year time horizons

To put the obtained results into perspective, additional analyses were carried out using time horizons from models that have previously analyzed the cost-effectiveness of the evaluated dressings [20,21].

The one-year time-horizon results obtained using our model indicated that the TLC-NOSF dressing is associated with a gain of 0.06 LYs _w/DFU_ DFU (0.24 versus 0.18 for the TLC-NOSF and control dressings, respectively), a gain of 0.01 QALYs (0.60 versus 0.59), a reduction of 0.005 in amputations (0.006 versus 0.011), and a total cost reduction of EUR 3,699 (EUR 40,182 versus EUR 43,881). The two-year time horizon results are reported in Table 9.

**Table 9:** Life years without DFU, quality-adjusted life years, number of amputations, and total costs from the scenario analysis (one patient, two-year time horizon, discounted outcomes).

	Life Years without DFU (LYs _w/DFU_)	Quality-Adjusted Life Years (QALYs)	Number Of Amputations	Total Costs (EUR)
**TLC-NOSF group (1)**	0.65	1.23	0.025	66,595
**Control group (2)**	0.42	1.20	0.031	79,980
**Difference (1)–(2)**	+0.23	+0.03	-0.006	-13,385
**ICER (EUR/LYs**_**w/DFU**_**)** *	Dominant			
**ICUR (EUR/QALYs)** *				

LYs_w/DFU_: life-years without DFU, QALYs: quality-adjusted life-years.

* Dominant means more LYs w/DFU/QALYs at a lower total cost.

## Discussion

The cost-effectiveness analysis that was performed here established that treating DFUs with TLC-NOSF dressings is more effective and less costly than treating them with neutral dressings, with magnitudes that are both medically relevant and economically significant from a French collective perspective. The robustness of the results was confirmed by the consistency of the better outcomes achieved with the TLC-NOSF dressings in all the sensitivity analyses. Moreover, while the TLC-NOSF strategy was always dominant, regardless of the duration of the wound at treatment initiation, the best outcomes were reported when the treatment was initiated as soon as possible [40].

Our economic model considered the specific features of DFU management in France. The clinical outcomes of the evaluated interventions and the population demographic characteristics were based on the European double-blind RCT EXPLORER [15], which included 19 French investigating centers. This RCT, published in *The Lancet Diabetes & Endocrinology*, demonstrated the superior clinical efficacy of the TLC-NOSF dressings in terms of the wound closure rate and time-to-closure in the management of patients with DFU. The high-quality of evidence of this RCT is acknowledged in recent systematic reviews [18,41] due to the double-blind design of the study, the highly relevant choice of the primary outcome, and the substantial and consistent benefits reported in the primary analysis, as well as in all the sensibility and sub-group analyses. The other inputs for our model were sourced from French databases and clinical studies relevant to the French perspective, considering notably the treatment of inpatients and outpatients in the French setting [28,32,33]. Potential differences in model inputs, compared to those of other models [24–28,42,43], were also assessed through deterministic and probabilistic sensitivity analyses. As illustrated in the tornado diagram, cost-utility plane, and cost-utility net monetary benefit curves, these input values may result in subsequent differences in costs per patient, but the results consistently point to better outcomes under the TLC-NOSF dressing strategy. The high level of confidence of the results is also supported by the fact that the probabilities of maximizing the net monetary benefit was consistently close to 1, regardless of the community's willingness to pay to gain 1 QALY for patients with DFUs.

This analysis, conducted from a French perspective, is the third to confirm the cost-effectiveness of TLC-NOSF dressings in DFU treatment after the ones conducted in the UK by the NICE [20] and in Germany by Lobmann et al. [21,22]. It also complements the evidence establishing the cost-effectiveness of the dressings in the management of VLU [20,44]. In each of these analyses, the TLC-NOSF strategy was established as a dominant one- and proven to be consistently more efficient and more cost-saving than neutral dressing. In fact, based on the cost analysis of its external assessment center [20], the NICE specified that using TLC-NOSF dressings to treat DFUs in the UK is associated with a cost saving of GBP 342 per patient after one year, and the analysis conducted from the statutory health insurance (SHI) perspective in Germany [21] showed a cost saving of EUR 2,566.52 per patient with a 100-week horizon. The one and two-year results of our cost-effectiveness model estimate cost savings per patient of EUR 3,699 and EUR 13,385.06, respectively. Comparison between models can be difficult and should be treated with caution. The magnitude of the potential savings is logically dependent on the parameters of the model and the perspective applied, normally according to the specifications of each country’s authorities. In this particular example, in addition to the different time horizons, the difference in perspective (collective perspective [all payers] versus single payer perspective [national health insurance]), the type of economic analysis (cost utility for France and the UK versus cost effectiveness for Germany), as well as the discounting methods, as recommended by the HAS [24], NICE [45], and IQWIG [46], generate expected differences in cost savings between countries. However, the use of TLC-NOSF dressings is always more cost-saving compared to the control, regardless of the model design.

The control dressing chosen in the EXPLORER study, and thus in this cost-effectiveness study, was a contact layer commonly used in DFU care [12]. Based on the conclusions of previous Cochrane and IWGDF systematic reviews [47,48], there was no evidence for differences between neutral wound dressings (such as hydrocolloid, alginate, foam, and contact layer) for any DFU outcome for people with diabetes treated in any setting. Therefore, the control dressing used in this study can be considered representative of any other type of neutral wound dressing available in the market.

Furthermore, this cost-effectiveness analysis is the first to establish the benefits of the TLC-NOSF dressing for a lifetime horizon. Each patient has been followed from the first dressing application until death, while health benefits and costs have been discounted at the rate of 2.5% per year according to the HAS recommendations [24]. The five health states considered in this study take into account the main complications that a DFU can encounter over time: infection, amputation, and death. By doing so, the model established that most of the savings are made on the costs associated with uninfected ulcers, which heal faster and therefore have fewer complications, hence the consequent savings on the infected state. Two amputations can also be avoided for every 100 patients treated with TLC-NOSF dressings—an outcome not only substantial in terms of cost savings, but also in terms of quality of life. The lack of a post-amputation state in our model could be considered a potential limitation, as it has been included in some other studies [21,28]. However, as TLC-NOSF dressings led to fewer amputations than neutral dressings, as also suggested by the NICE model, this structure is conservative for the assessed dressings.

From an economic viewpoint, our analysis also confirmed that best outcomes could be achieved when the dressings are used as a first-line treatment compared to neutral dressings, as previously demonstrated in the German perspective [22]. It should be reassuring for decisionmakers to know that these results are consistent with the clinical ones reported in real-life studies [16] as in clinical trials not only regarding DFUs but also leg and pressure ulcers [15,17,49]. In France, two different committees assess, independently and in parallel, the clinical effectiveness and cost effectiveness of medical devices. For the TLC-NOSF French dossier, an additional budget impact analysis (BIA) was performed. The base case results showed that routine use of TLC-NOSF dressings, compared to the same dressings without NOSF, induced a mean saving of EUR 3,345 per patient per year (over a four-year time horizon) for the mandatory health insurance (MHI). Cost savings were consistently reported regardless of the wound duration at treatment initiation but reached the highest amount when wounds with a duration of two months or less were treated (EUR 4,771 per patient per year). Considering that around 80,000 patients with DFUs in France could benefit from this treatment strategy including TLC-NOSF dressings, the potential cost savings could reach the substantial amount of EUR 267.6 million per year. As treating patients with TLC-NOSF dressings requires no specific training or additional workload from healthcare professionals nor constraints for the patients, a wide implementation in the community and hospitals should be easily manageable. The generated savings could then be reallocated to support prevention programs including comprehensive foot care and patient education among those at moderate/high risk of developing foot ulcers or to the development of telemedicine, for examples. In the current COVID-19 pandemic context, patients with diabetes are known to be at high risk to develop severe form of the coronavirus disease and hospital beds are counted. More than ever, effective treatment that can reduce the risk of complications and hospital stays for these patients would ultimately have an enormous impact.

## Conclusions

The cost effectiveness and cost utility of TLC-NOSF dressings were established from a French collective perspective compared to those of neutral dressings. Treating DFUs with TLC-NOSF dressings is more effective and more cost-saving than treating them with neutral dressings. The robustness of the results was confirmed by the consistency of the better outcomes achieved when using TLC-NOSF dressings in all analyses, and the results complement the cost effectiveness analyses previously conducted for other countries. Moreover, while the TLC-NOSF strategy was always dominant, regardless of the duration of the wounds at treatment initiation, the subgroup analyses highlighted that the DFU duration at baseline is a discriminant factor, and the best outcomes were reported when treatment was initiated as soon as possible. These results can be used to guide healthcare decisionmakers, and its use as first-line treatment versus neutral dressings can lead to benefit maximization.

## Supporting information

S1 File Input data populating the cost-effectiveness model and hypotheses. (XLSX)

S2 File Simulated data (Markov traces of the cost-effectiveness model for the last simulated individual). (XLSX)
